# Kinetics and Connectivity Properties of Parvalbumin- and Somatostatin-Positive Inhibition in Layer 2/3 Medial Entorhinal Cortex

**DOI:** 10.1523/ENEURO.0441-21.2022

**Published:** 2022-02-15

**Authors:** Fernando R. Fernandez, Guillem Via, Carmen C. Canavier, John A. White

**Affiliations:** 1 Department of Biomedical Engineering, Center for Systems Neuroscience, Neurophotonics Center, Boston University, Boston, Massachusetts 02215; 2 Department of Cell Biology and Anatomy, Louisiana State University Health Sciences Center, New Orleans, Louisiana 70112

**Keywords:** gap, inhibition, junctions, kinetics, medial entorhinal cortex, synapse

## Abstract

Parvalbumin-positive (Pvalb^+^) and somatostatin-positive (Sst^+^) cells are the two largest subgroups of inhibitory interneurons. Studies in visual cortex indicate that synaptic connections between Pvalb^+^ cells are common while connections between Sst^+^ interneurons have not been observed. The inhibitory connectivity and kinetics of these two interneuron subpopulations, however, have not been characterized in medial entorhinal cortex (mEC). Using fluorescence-guided paired recordings in mouse brain slices from interneurons and excitatory cells in layer 2/3 mEC, we found that, unlike neocortical measures, Sst^+^ cells inhibit each other, albeit with a lower probability than Pvalb^+^ cells (18% vs 36% for unidirectional connections). Gap junction connections were also more frequent between Pvalb^+^ cells than between Sst^+^ cells. Pvalb^+^ cells inhibited each other with larger conductances, smaller decay time constants, and shorter delays. Similarly, synaptic connections between Pvalb^+^ and excitatory cells were more likely and expressed faster decay times and shorter delays than those between Sst^+^ and excitatory cells. Inhibitory cells exhibited smaller synaptic decay time constants between interneurons than on their excitatory targets. Inhibition between interneurons also depressed faster, and to a greater extent. Finally, inhibition onto layer 2 pyramidal and stellate cells originating from Pvalb^+^ interneurons were very similar, with no significant differences in connection likelihood, inhibitory amplitude, and decay time. A model of short-term depression fitted to the data indicates that recovery time constants for refilling the available pool are in the range of 50–150 ms and that the fraction of the available pool released on each spike is in the range 0.2–0.5.

## Significance Statement

Two large and distinct classes of interneurons in medial entorhinal cortex (mEC) include parvalbumin-positive (Pvalb^+^) and somatostatin-positive (Sst^+^) cells. Previous work has demonstrated unique functions with regard to spatial tuning and network oscillations for these two interneuron populations. Potential differences in kinetics of inhibition and likelihood of connection from these two interneuron groups, however, have not been quantified. Here, using fluorescence to guide intracellular recordings, we quantified the synaptic connections from both types of interneurons. We indicate that Sst^+^ and Pvalb^+^ express different synaptic kinetics that are target cell specific. In contrast to neocortical measures, we find substantial connections between Sst^+^ interneurons.

## Introduction

Medial entorhinal cortex (mEC) plays a significant role in spatial navigation (Burgalossi and Brecht, 2014; Sasaki et al., 2015). In layer 2/3 mEC, the neurophysiological correlates of this role are partially supported by spatially tuned cells (“grid cells”) that generate spikes at the vertices of a hexagonal grid formed during movement of an animal (Fyhn et al., 2004; Hafting et al., 2005). The region also generates theta-nested gamma frequency oscillations (Colgin et al., 2009) that synchronize grid cell spiking to specific phases of a network-wide theta oscillation (Hafting et al., 2008; Reifenstein et al., 2012).

Both spatial tuning and oscillations in mEC are often accounted for using a canonical circuit composed of excitatory and inhibitory cells connected through recurrent excitation and negative feedback (Shipston‐Sharman et al., 2016). In mEC, fast-firing interneurons participate in theta-nested gamma oscillations through a mechanism similar to pyramidal (Pyr) interneuron gamma oscillations in cortex and hippocampus (Pastoll et al., 2013); by inhibiting stellate cells, these neurons set the phase of spiking and frequency of gamma oscillations during network activation. Fast-firing interneurons also provide the sole synaptic communication path between stellate cells, which lack recurrent excitatory connections (Couey et al., 2013; Pastoll et al., 2013; but see Fuchs et al., 2016). In contrast, a subset of low threshold-spiking interneurons have been shown to suppress network oscillations in mEC (de Filippo et al., 2021). Further, in behaving animals, inhibition from layer 2/3 parvalbumin-positive (Pvalb^+^) and somatostatin-positive (Sst^+^) interneurons have separate roles in setting the spatial tuning and firing rates of layer 2 grid cells (Miao et al., 2017).

In cortex and hippocampus, Pvalb^+^ and Sst^+^ interneurons comprise two families of interneurons that, to a first approximation, correspond to fast-firing and low threshold-spiking interneurons, respectively (Rudy et al., 2011; Kepecs and Fishell, 2014; Tremblay et al., 2016; Pelkey et al., 2017). Across different regions, fast-acting, perisomatic-targeting inhibitory feedback typically originates from fast-firing Pvalb^+^ cells (Kawaguchi and Kubota, 1997, 1998; Kubota et al., 2016). These cells are highly interconnected through synapses and gap junctions (Bartos et al., 2002; Hjorth et al., 2009). Conversely, Sst^+^ interneurons are more diverse electrophysiologically (Tremblay et al., 2016; Yavorska and Wehr, 2016), target dendrites (Kawaguchi and Kubota, 1998), can be electrical coupled (Amitai et al., 2002; Fanselow et al., 2008), and provide inhibition onto other, non-Sst^+^, neurons, with little evidence of synaptic connections between Sst^+^ cells (Pfeffer et al., 2013). Specifically, measures in visual cortex indicate a lack of synaptic connections between Sst^+^ interneurons (Pfeffer et al., 2013).

To date, the likelihood and synaptic kinetics of inhibition from Pvalb^+^ and Sst^+^ interneurons in mEC have not been measured. Although some properties are likely shared with other brain regions, significant differences have been observed between regions (Tremblay et al., 2016; Yavorska and Wehr, 2016). Measures in mEC, therefore, can guide specific mechanisms and models of mEC activity with regard to the role of inhibition in spatial tuning and network synchrony. Using Cre-based expression of the tdTomato fluorophore, we targeted Pvalb^+^ and Sst^+^ interneurons and used paired recordings from mouse slices to establish the properties of inhibition, both between interneurons as well as onto excitatory cells, in layer 2/3 mEC.

## Materials and Methods

### Ethics statement

All experimental protocols were approved by the Boston University Institutional Animal Care and Use Committee.

### Transgenic mice

To target Pvalb^+^ and Sst^+^ interneurons, we used mice expressing the red fluorescent protein tdTomato in either interneuron population. For Pvalb^+^ neurons, C57BL/6J background, Pvalb-Cre mice (Taniguchi et al., 2011; stock #017320, The Jackson Laboratory) were crossed with the lox-stop-lox tdTomato reporter mice (Zariwala et al., 2011; stock #007914, The Jackson Laboratory). The same reporter mouse was crossed to Sst-Cre mice (Taniguchi et al., 2011; stock # 013044, The Jackson Laboratory) to visualize Sst^+^ interneurons.

### Slice preparation

Horizontal slices of entorhinal cortex and hippocampus were prepared from 2- to 8-month-old mice of either sex. After anesthetization with isoflurane and decapitation, brains were removed and immersed in 0°C sucrose-substituted artificial CSF (ACSF) consisting of the following (in mm): sucrose 185, KCl 2.5, NaH_2_PO_4_ 1.25, MgCl_2_ 10, NaHCO_3_ 25, glucose 12.5, and CaCl_2_ 0.5. Slices were cut to a thickness of 400 µm with a vibratome (model VT1200, Leica Microsystems). Slices were then incubated at 35°C for 20 min in ACSF consisting of the following (in mm): NaCl 125, NaHCO_3_ 25, d-glucose 25, KCl 2, CaCl_2_ 2, NaH_2_PO_4_ 1.25, and MgCl_2_ 1. Afterward, slices were cooled to room temperature (20°C). After the incubation period, slices were moved to the stage of a two-photon imaging system (Thorlabs) with a mode-locked Ti:Sapphire laser (Chameleon Ultra II, Coherent) set to wavelengths between 915 and 950 nm, which was used to excite both Alexa Fluor 488 and tdTomato using a 20×, numerical aperture 1.0 (Olympus) objective lens. Laser scanning was performed using resonant scanners and fluorescence was detected using two photo-multiplier tubes (Hamamatsu) equipped with red and green filters to separate emission from Alexa Fluor 488 and tdTomato. The stage of the microscope contained recirculating ASCF, with all recordings conducted between 34°C and 36°C.

### Electrophysiology

Electrodes were pulled using a horizontal puller (Sutter Instrument) using filament, thin-wall glass (Sutter Instrument). Intracellular pipette solution consisted of the following (in mm): K-gluconate 120, KCl 20, HEPES 10, diTrisPhCr 7, Na_2_ATP 4, MgCl_2_ 2, Tris-GTP 0.3, and EGTA 0.2, and buffered to pH 7.3 with KOH. To visualize electrodes, the cyan-green fluorescent dye Alexa Fluor 488 hydrazide (Thermo Fisher Scientific) was added to the intracellular electrode solution (0.3% w/v). To patch nonfluorescent, excitatory cells, we used a “shadow” patch technique (Kitamura et al., 2008) in which extracellular green fluorescence contrast with cells that do not fluorescence. Although this does not exclude the possibility of recording from interneurons, the values of spike half-width in probable excitatory cells indicated little overlap with those of Pvalb^+^ and Sst^+^ neurons.

Electrode resistances were between 4 and 7 MΩ, with access resistance values between 15 and 38 MΩ. Seal resistance values were always >2 GΩ. Capacitance was fully compensated in voltage clamp during the on-cell configuration before breaking into the cell. For current-clamp recordings, full bridge balance compensation was used. Series resistance compensation between 45% and 65% was used during voltage-clamp recordings. Voltage trace signals were amplified and low-pass filtered at 10–20 kHz before being digitized at 20–50 kHz. For current traces, signals were low-pass filtered at 4 kHz. All electrophysiology was conducted using a Multiclamp 700B Microelectrode Amplifier (Molecular Devices) and a Digidata 1550 Data Acquisition System (Molecular Devices). Liquid junction potentials were not corrected.

### Recording protocols

A series of 1-s-long hyperpolarizing and depolarizing current pulses were used to generate spike frequency–current relationships. Spike half-width was taken from the first current pulse that generated spikes. Using the same data, the membrane decay time constant was acquired using an exponential fit to the voltage. To determine the presence of a synaptic connection, the postsynaptic cell was depolarized to −40 mV, while driving the presynaptic cell with a brief (2 ms), strong (>0.5 nA) pulse that drove a single spike. For measures of excitatory synaptic connections on interneurons, cells were voltage clamped at −70 mV. For frequency-dependent synaptic depression measures, pulses were delivered at 5, 10, 20, 50, 100, and 200 Hz. Synaptic current responses were averaged across 25–50 trials. For gap junction measures, a square pulse or spike was generated in the presynaptic cell and 25–50 trials were averaged in the postsynaptic cell. A measured junction potential of ∼11 mV was not subtracted from recordings. Recordings were taken from slices between 3.2 and 4.3 mm from the dorsal surface (bregma) of the brain.

### Data analyses

For current-clamp analyses of spike shape, spike threshold was defined using the peak of the second derivative of the spike waveform. Spike half-width was taken as the width of the spike at voltages corresponding to the half-amplitude (mid-point between spike peak amplitude and threshold). For time constant measures, a current pulse was used to depolarized cells to a value slightly below spike threshold. A single exponential decay function was used to fit the membrane voltage time course associated with hyperpolarization and the return to resting voltage resulting from the end of the current pulse.

Averaged individual postsynaptic current decay and rise time courses were fit with single exponential functions. Synaptic delay was measured as the time between spike threshold and the 10% rise time of the averaged synaptic current response. All peak amplitudes were taken as the peak of the averaged synaptic current response. In a subset of recordings, where individual responses were large, we compared the averaged response to the distribution across trials. In this dataset, mean responses sat near the center of the trial-to-trial distribution, suggesting that averaging did not impact our estimates of rise kinetics and delays.

The probability of chemical synaptic connectivity was calculated assuming that the probability of connections in each direction between a pair of neurons were equal and independent.

### Statistical analyses

All values are presented as the mean along with the SD. The normality of data points was established using a Shapiro–Wilk and Lilliefors test. A positive result (*p* < 0.05) from either of these tests was used to determine normality, and the use of nonparametric statistical tests is noted in the Results section. For non-normally distributed data points, results are presented as the median along with the first quartile (Q1) and third quartile (Q3) values.

### Modeling

The model by Markram and Tsodyks (1996) predicts the amplitude of inhibitory currents from a differential equation for the available fraction of transmitter *x* that evolves according to the following:

$$\frac{dx}{dt}=\frac{1-x}{\tau_{r}}-U_{\text{SE}}x\delta \left(t-t_{s}\right),$$

where $t_{s}$ is the spike time, $\tau_{r}$ is the recovery time to replenish the available pool of vesicles for release, *U*_SE_ is the fraction of available pool released by each spike, and the value of x before a spike is proportional to the peak current value of the IPSC. The solution to the differential equation between presynaptic spikes is $x(t)=1-(1-x_{i})e^{-\frac{(\text{t}-t_{s)}}{\tau_{r}}}$. However, there is a discontinuous decrement, *x*, by an amount *U*_SE_ *x* after each spike, so a discrete map from spike to spike is required, as follows:

$$x_{i}=1-(1-x_{i-1}(1-U_{\text{SE}}))e^{\frac{-(t-t_{s})}{\tau_{r}}},$$

where *i* denotes presynaptic spike number. Normalizing the spike amplitude so that the initial value of *x* is 1 and substituting 1 over the frequency of the presynaptic spike train for *t – t_s_*, we can write this recursively as follows:

$$x_{k}=\left(1-e^{-\frac{1}{f\tau_{r}}}\right)\frac{1-{\left[\left(1-U_{\text{SE}}\right)e^{-\frac{1}{f\tau_{r}}}\right]}^{k}}{1-\left(1-U_{\text{SE}}\right)e^{-\frac{1}{f\tau_{r}}}}+{\left[(1-U_{\text{SE}})e^{-\frac{1}{f\tau_{r}}}\right]}^{k}.$$

We fit each experimental train of the normalized inhibitory peak amplitudes to the expression above by adjusting the two parameters *U*_SE_ and $\tau_{r}$ to minimize the least squared error using *curve_fit* from *scipy.optimize*. If the *R*^2^ for the linear regression between each train and the predicted values from the best fit was >0.8, the trace was used in the calculation of the mean of the parameter values. Smaller values indicated a noisy trace, likely from a small synapse. Maximum likelihood estimation of the parameters yielded similar values, and the datasets passed a Shapiro–Wilcox test for normality. The Akaike information criterion and rms yielded similar results to *R*^2^, but we chose to use *R*^2^ to accept or reject datasets because the values are constrained (between 0 and 1) whereas the other metrics are not. The expression above sufficed for the connections between interneurons (Sst–Sst and Pvalb–Pvalb). The fit to the data from synapses onto the excitatory cells were improved by adding a correction for temporal summation from up to three previous spikes $x_{k-j}e^{-\frac{j}{f\tau_{r}}}$, where *j* is the index of whether the contribution is from the immediately preceding spike (*j* = 1) or farther back in the spike train.

The previously measured time constant for synaptic decay at each synapse was used rather than fitting an additional unknown parameter.

## Results

### High degree of connectivity between mEC Pvalb^+^ interneurons

To address the likelihood of connections and synaptic kinetics of inhibition from layer 2/3 Pvalb^+^ and Sst^+^ interneurons, we performed dual intracellular patch recordings in mice expressing the tdTomato fluorophore in Pvalb^+^ or Sst^+^ cells. We targeted both fluorescent and nonfluorescent cells and used intracellular electrophysiological measures to distinguish between subtypes of nonfluorescent cells (pyramidal and stellate cells) in layer 2.

**Figure 1.**
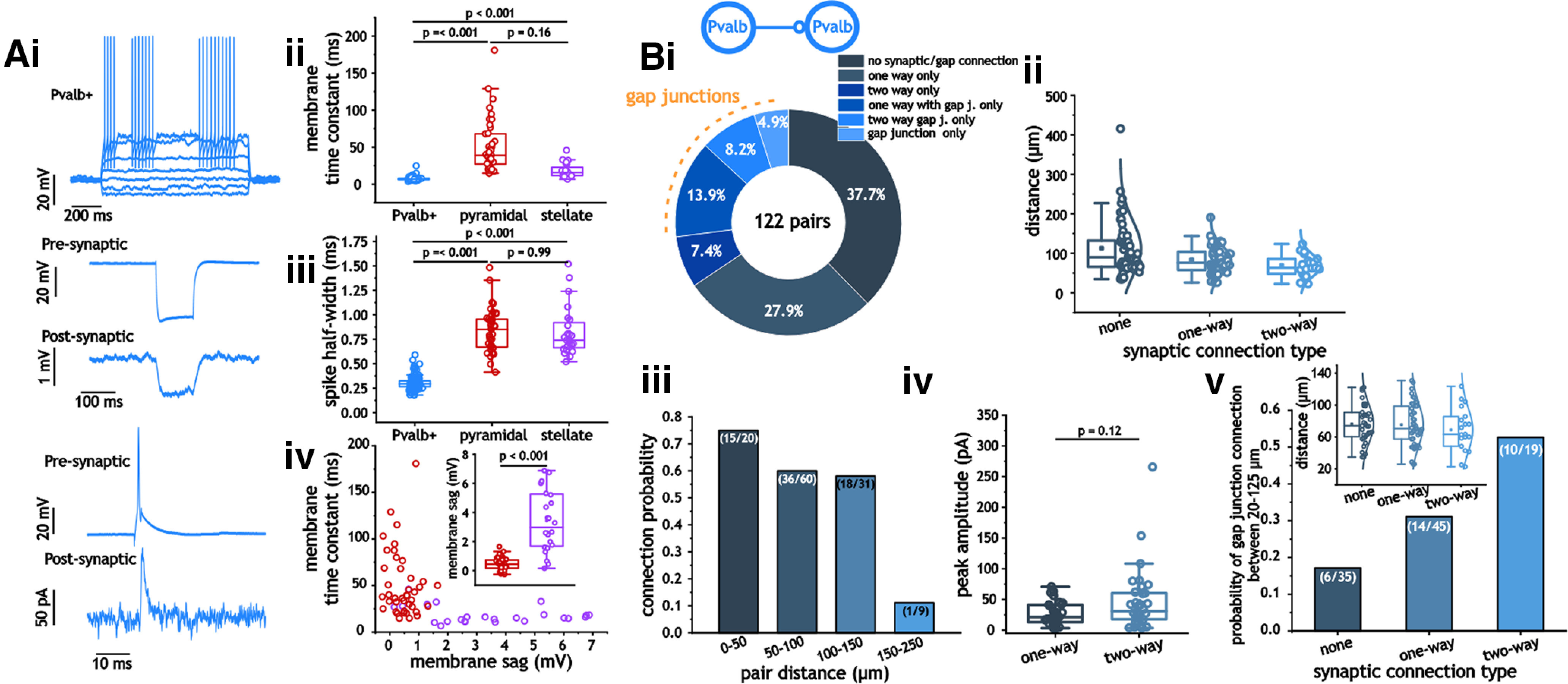
Synaptic and gap junction connections between Pvalb^+^ interneurons. ***Ai–iv***, Example traces from Pvalb^+^ cells in response to current steps, along with examples of gap junction and synaptic connections (***i***). Membrane time constant (***ii***) and spike half-width (***iii***) measures in Pvalb^+^, pyramidal, and stellate cells, along with a plot of membrane voltage sag and time constant in pyramidal and stellate cells (***iv***). ***Bi***, Distribution and probability of connection types between Pvalb^+^ interneurons. Pie chart indicating the distribution of connection types between Pvalb^+^ interneurons. ***Bii***, ***iii***, Plots of pair distance as a function of synaptic connection type (none, one-way, two-way; ***ii***) and connection probability of pairs between 20 and 250 μm (***iii***). ***Biv***, Peak inhibitory synaptic amplitude in one-way and two-way connected Pvalb^+^ interneurons. ***Bv***, Plot of gap junction probability in unconnected, one-way, and two-way connected Pvalb^+^ interneuron pairs at distances between 20 and 125 μm.

Aside from the fluorescent marker, Pvalb^+^ cells could be differentiated from excitatory cells due a much smaller membrane time constant [6.8 ms (Q1, 3.72; Q3, 8.0); *p* < 0.001, Kruskal–Wallis ANOVA; Fig. 1*Aii*] and a spike half-width [0.29 ms (Q1, 0.27; Q3, 0.32); *p* < 0.001, Kruskal–Wallis ANOVA;  Fig.1*Aiii*], which is consistent with the fast-firing phenotype. In addition, we distinguished between layer 2 pyramidal and stellate cells using the membrane overshoot in response to a negative current pulse associated with the expression of a hyperpolarizing-activated cation current (*I*_H_) and the membrane decay time constant; stellate cells expressed significantly larger membrane overshoots in response to a hyperpolarizing current (3.43 ± 2.13 vs 0.45 ± 0.44 mV; *p* < 0.001, two-way Student’s *t* test;  Fig. 1*Aiv*). Combined with a smaller membrane time constant, the two factors could be used to differentiate most stellate cells from pyramidal cells (Fig. 1*Aiv*). In cases where neither of these two factors was applicable, we used the presence of perithreshold oscillations to distinguish cells (only used in five cells). To detect synapses and gap junctions, we averaged between 25 and 50 sweeps in voltage clamp (held at −40 mV) while driving a spike using a brief current pulse (2 ms) in the other neuron.

At pair distances between 0 and 150 μm, we observed a high probability of connection between mEC Pvalb^+^ interneurons. Over all distances tested, we found that 76 of 122 pairs expressed a connection either in the form of a chemical synapse (43 of 122), a gap junction (6 of 122), or both (27 of 122; Fig. 1*Bi*); therefore, the probability of chemical synaptic connectivity was 35.7% (87 of 244) in each direction, and the probability of bidirectional gap junctional connectivity was 27% (33 of 122). At distances >150 μm, connection probability (of either type) fell sharply (Fig. 1B*iii*), which is consistent with the extent of axonal arborization of Pvalb^+^ interneurons within layer 2/3 (Martínez et al., 2017; Grosser et al., 2021). We also found that the synaptic amplitude for unidirectionally connected pairs [21.4 pA (Q1, 12.9; Q3, 41.0)] did not differ significantly from the size of synapses present in bidirectionally connected pairs [30.9 pA (Q1, 17.8; Q3, 60.7); *p* = 0.12, Mann–Whitney test; Fig. 1*Biv*]. Using a step depolarization between 10 and 20 mV in one cell, we measured the strength of gap junction connections between Pvalb^+^ cells as the ratio of the two voltage responses. This value was 0.03 (Q1, 0.02; Q3, 0.06) across 17 measured pairs.

Next, the prevalence of gap junctions was measured between synaptically connected and unconnected pairs. In our dataset, synaptically unconnected pairs occurred at longer pair distances than those of unidirectional or bidirectional connections (Fig. 1*Bii*). To eliminate this feature from artificially lowering the prevalence of gap junctions in synaptically unconnected pairs, we limited our analysis to pairs at distances between 20 and 125 μm. Under this constraint, the pair distances for all three categories of synaptic connections were statistically similar (*p* = 0.57, one-way ANOVA; no connection, 76.4 ± 23.9 μm; unidirectional, 75.4 ± 26.8 μm; bidirectional, 68.9 ± 27.3 μm; Fig. 1*Bv*, inset). Using this dataset, we found that the probability of gap junctions was significantly greater in bidirectionally (0.53 vs 0.17; *p* = 0.01, two-sided Fisher’s exact test), but not unidirectionally connected pairs when compared with unconnected pairs (Fig. 1*Bv*).

### Connectivity and kinetics between mEC Pvalb^+^ interneurons and excitatory neurons

**Figure 2.**
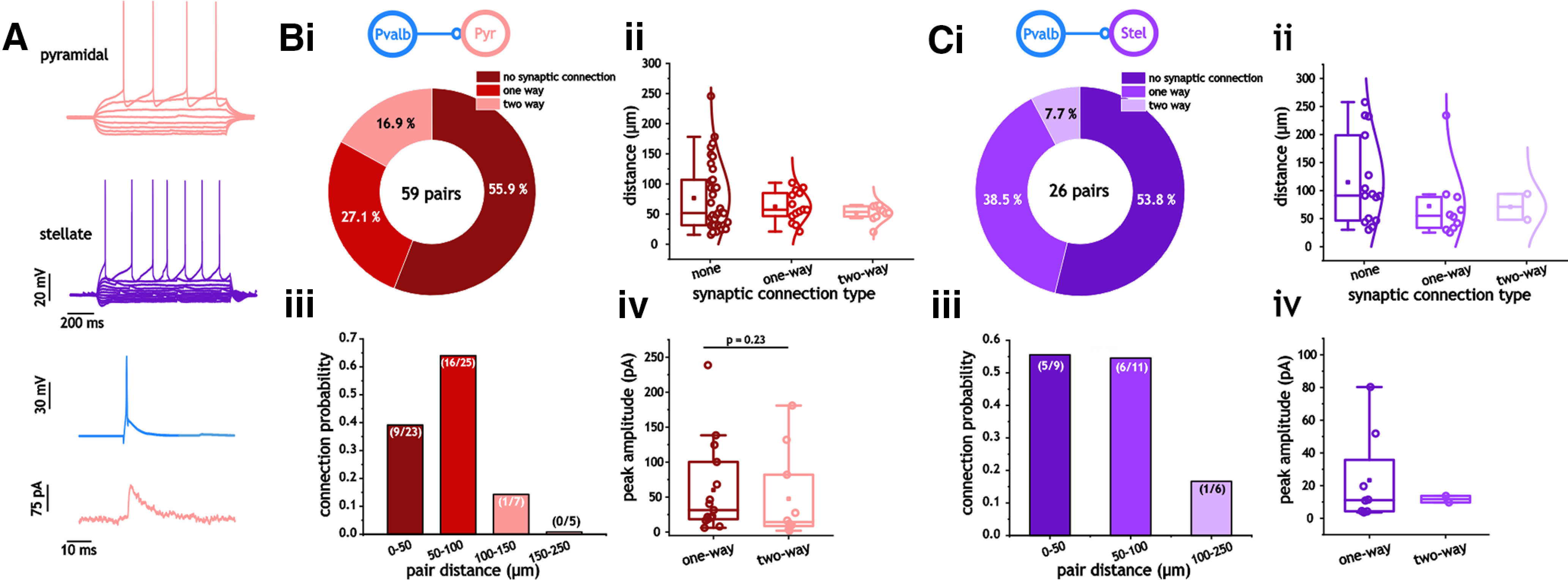
Synaptic connections between Pvalb^+^ interneurons and excitatory cells. ***A***, Distribution of connections between Pvalb^+^ and pyramidal cells. ***Bi***, Pie chart indicating the distribution of connection types between Pvalb^+^ and pyramidal cells. ***Bii***, ***iii***, Plots of pair distance as a function of synaptic connection type (none, one-way, two-way; ***ii***) and connection probability between 20 and 250 μm (***iii***). ***Biv***, Peak inhibitory synaptic amplitude in one-way and two-way connected Pvalb^+^ interneurons. ***C***, Distribution of connections between Pvalb^+^ and pyramidal cells. ***Ci***, Pie chart indicating the distribution of connection types between Pvalb^+^ and stellate cells. ***Cii***, ***iii***, Plots of pair distance as a function of synaptic connection type (none, one-way, two-way; ***ii***) and connection probability between 20 and 250 μm (***iii***). ***Civ***, Peak inhibitory synaptic amplitude in one-way and two-way connected Pvalb^+^ interneurons.

We conducted a similar set of analyses for connections between Pvalb^+^ and excitatory cells (I_Pvalb_–E; Fig. 2*A*). Unlike interneuron pairs, we never observed gap junctions in I_Pvalb_–E cell pairs. Connection likelihood was also generally lower for I_Pvalb_–E pairs compared with I_Pvalb_–I_Pvalb_ pairs. Including both excitatory cell types, 38 of 85 I_Pvalb_–E pairs were connected compared with 76 of 122 I_Pvalb_–I_Pvalb_ pairs (*p* = 0.02, two-sided Fisher’s exact test). There were 26 unidirectional connections and 12 bidirectional connections; therefore, the probability of chemical synaptic connectivity was 29.4% (50 of 170) in each direction. Both pyramidal and stellate cells expressed statistically similar ratios of unconnected and connected pairs (33 of 26 vs 14 of 12; *p* = 0.99, two-sided Fisher’s exact test; Fig. 2*Bi*,*Ci*) For both cell types, connection probability also dropped off at distances >100 μm (Fig. 2*Biii*,*Ciii*). Like I_Pvalb_–I_Pvalb_ pairs, we found no significant difference in the size of inhibitory synapses between unidirectionally and bidirectionally connected pairs of Pvalb^+^ and pyramidal (I_Pvalb_–E_Pyr_) cells [31.1 pA (Q1, 18.4; Q3, 100.2) vs 14.2 pA (Q1, 7.6; Q3, 94.4); *p* = 0.23, Mann–Whitney test; Fig. 2*Biv*); we did not have enough bidirectionally connected Pvalb^+^ stellate (I_Pvalb_–E_Stel_) cell pairs to test potential differences in this measure (Fig. 2*Civ*).

**Figure 3.**
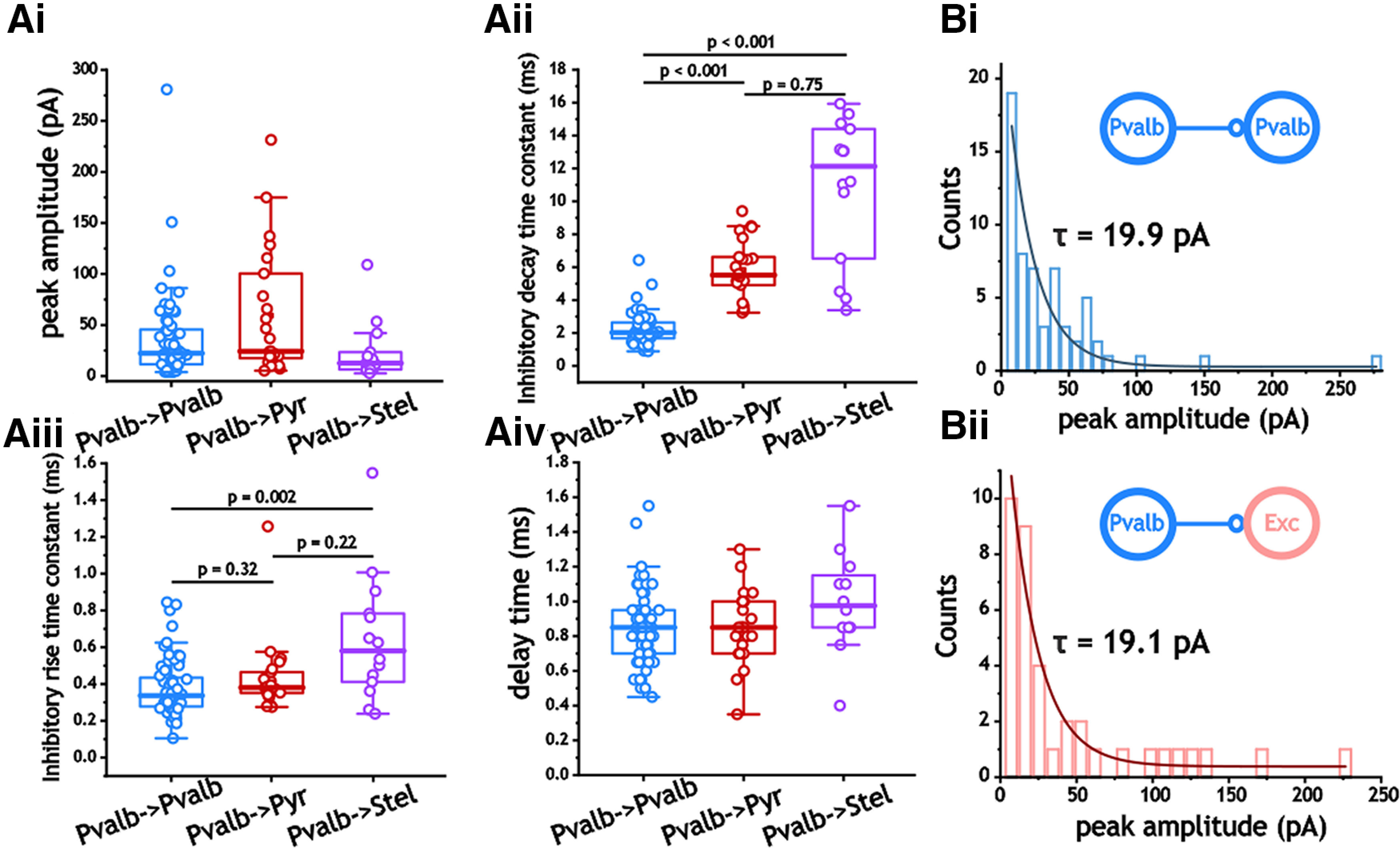
Synaptic inhibitory properties from Pvalb^+^ interneurons. ***Ai–iv***, Comparison of peak amplitude (***i***), inhibitory decay time constant (***ii***), inhibitory rise time constant (***iii***), and delay time (***iv***). ***B***, Distributions of inhibitory synaptic amplitude in Pvalb^+^ interneurons and excitatory cells (stellate and pyramidal cells were pooled together). Histograms were fit with a single exponential function.

The kinetics of inhibition indicated clear differences among the three cell types. We focused on amplitude, decay time, delay, and rise time as these properties are crucial in many models of inhibitory activity with regard to synchrony and oscillations (Bartos et al., 2007; Economo and White, 2012; Keeley et al., 2017). The largest difference among these parameters was the inhibitory decay time constant (single exponential fit; Kruskal–Wallis ANOVA, *p* < 0.001; Fig. 3*Aii*). Synaptic inhibition decayed much faster in Pvalb^+^ cells than in either pyramidal or stellate cells [2.0 ms (Q1, 1.7; Q3, 2.6) vs 5.5 ms (Q1, 4.9; Q3, 6.6) and 12.1 ms (Q1, 6.0; Q3, 14.5); *p* < 0.001, Dunn’s test; Fig. 3*Aii*). Although generally larger, the difference in decay time constants between stellate and pyramidal cells did not reach the significance threshold (*p* = 0.75, Dunn’s test; Fig. 3*Aii*).

We also found differences in the rise time constant between I_Pvalb_–I_Pvalb_ and stellate cell pairs (*p* = 0.002; Kruskal–Wallis ANOVA), with stellate cells showing larger rise time constants [0.34 ms (Q1, 0.28; Q3, 0.44) vs 0.58 ms (Q1, 0.40; Q3, 0.81); *p* = 0.002; Fig. 3*Aiii*]. In contrast, neither delay time (*p* = 0.12, one-way ANOVA) nor peak amplitude (*p* = 0.06, Kruskal–Wallis ANOVA) differed among the three pair categories (Fig. 3*Ai*,*iv*). Consistent with previous measures of excitatory (Song et al., 2005) and inhibitory (Bartos et al., 2001) synapses in other regions, the amplitude of inhibitory synapses expressed strong, non-Gaussian distributions (Shapiro–Wilk and Lilliefors test, *p* < 0.001). Distributions of I_Pvalb_–I_Pvalb_ and I_Pvalb_–E cell pair synaptic amplitudes fit an exponential decay and were dominated by small synapses accompanied by fewer, larger synapses (Fig. 3*Bi*,*ii*).

### Connectivity and kinetics between mEC Sst^+^ interneurons

**Figure 4.**
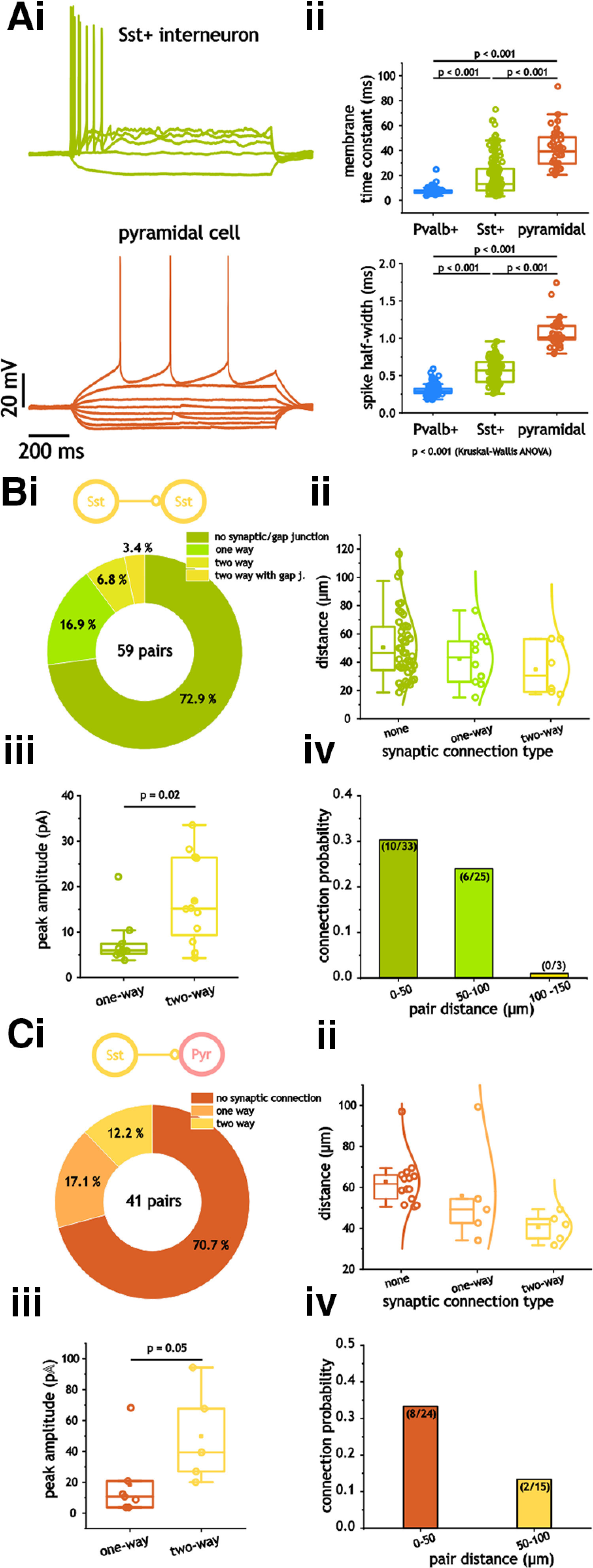
Synaptic and gap junction connections from Sst^+^ interneurons. ***Ai, ii***, Membrane time constant (***i***) and spike half-width (***ii***) measures in Sst^+^ and pyramidal cells. ***B***, Distribution of connection types and probability between Sst^+^ interneurons. ***Bi***, Pie chart indicating the distribution of connection types between Sst ^+^ and pyramidal cells. ***Bii***, ***iii***, Plots of pair distance as a function of synaptic connection type (none, one-way, two-way; ***ii***) and connection probability between 20 and 150 μm (***iii***). ***Biv***, Peak inhibitory synaptic amplitude in one-way and two-way connected Sst^+^ interneurons. ***C***, Distribution of connections between Sst^+^ and pyramidal cells. ***Ci***, Pie chart indicating the distribution of connection types between Sst ^+^ and pyramidal cells. ***Cii***, ***iii***, Plots of pair distance as a function of synaptic connection type (none, one-way, two-way; ***ii***) and connection probability between 20 and 150 μm (***iii***). ***Civ***, Peak inhibitory synaptic amplitude in one-way and two-way connected Sst^+^ interneurons.

Next, we focused on inhibition from Sst^+^ interneurons. As with Pvalb^+^ neurons, Sst^+^ neurons could be differentiated from pyramidal cells through their membrane decay time constant and spike half-width (Fig. 4*A*). Although not as small as Pvalb^+^ neurons, both membrane time constant (13.1 ms [Q1, 7.9; Q3, 25.3] vs 39.2 ms (Q1, 29.0; Q3, 51.0)] and spike half-width [0.57 ms (Q1, 0.41; Q3, 0.68) vs 1.0 ms (Q1, 0.98; Q3, 1.2)] were significantly smaller than those of pyramidal cells (*p* < 0.001, Dunn’s test; Fig. 4*Aii*).

Over all distances tested, we found that 16 of 59 Sst^+^ pairs expressed a connection either in the form of a chemical synapse (14 of 59) or a gap junction (2 of 59; Fig. 4*Bi*). Compared with I_Pvalb_–I_Pvalb_ pairs, synaptic and gap connections between I_Sst_–I_Sst_ neurons were far less likely (*p* < 0.001, Fisher’s exact test). Crucially, because of the lack of connections at distances >100 μm (Fig. 4*Biv*), our average I_Sst_–I_Sst_ pair distance was shorter than that for I_Pvalb_–I_Pvalb_ pairs [46.7 μm (Q1, 28.9; Q3, 65.0) vs 81.3 μm (Q1, 61.9; Q3, 116)]. Thus, despite shorter pair distances, connection probabilities were still far lower than those of I_Pvalb_–I_Pvalb_ pairs. We also found that the amplitude of synapses between I_Sst_–I_Sst_ pairs was significantly different depending on whether cells were unidirectionally or bidirectionally connected [6.0 pA (Q1, 5.2; Q3, 8.1) vs 15.1 pA (Q1, 8.6; Q3, 26.4; *p* = 0.02, Mann–Whitney test; Fig. 4*Biii*). Unlike I_Pvalb_–I_Pvalb_ pairs, gap junctions in I_Sst_–I_Sst_ pairs were observed in only two pairs, both of which also had bidirectional synaptic connections.

### Connectivity and kinetics between mEC Sst^+^ interneurons and pyramidal cells

For I_Sst_–E pairs, we limited our measures to pyramidal cells as we only observed three pairs containing stellate cells, with only 1 of these pairs being connected. This is likely because of the deeper location of Sst^+^ neurons within layer 2/3 (Tremblay et al., 2016), as well as lower overall density across cortical layers (Amitai et al., 2002). This is also consistent with optogenetic experiments indicating greater connectivity between Sst^+^ and pyramidal cells (Kecskés et al., 2020). In contrast to I_Sst_–I_Sst_ pairs, connection probabilities between I_Sst_ and E_Pyr_ were not significantly lower than those measured in I_Pvalb_–E pairs (12 of 41 vs 38 of 85; *p* = 0.12, two-sided Fisher’s exact test). If, however, we limited our connection measures in I_Pvalb_–E pairs to a range similar those of I_Sst_–E_Pyr_ pairs (20–125 μm), connection probability was indeed lower (12 of 41 vs 37 of 73, *p* = 0.03; two-sided Fisher’s exact test). The amplitude of synapses between I_Sst_–E_Pyr_ pairs also seemed to depend on whether cells were unidirectionally or bidirectionally connected. The difference, however, was at the margin of statistical significance [10.7 pA (Q1, 3.7; Q3, 20.9) vs 39.4 pA (Q1, 23.5; Q3, 81.0); *p* = 0.05, Mann–Whitney test; Fig. 4*Ciii*).

**Figure 5.**
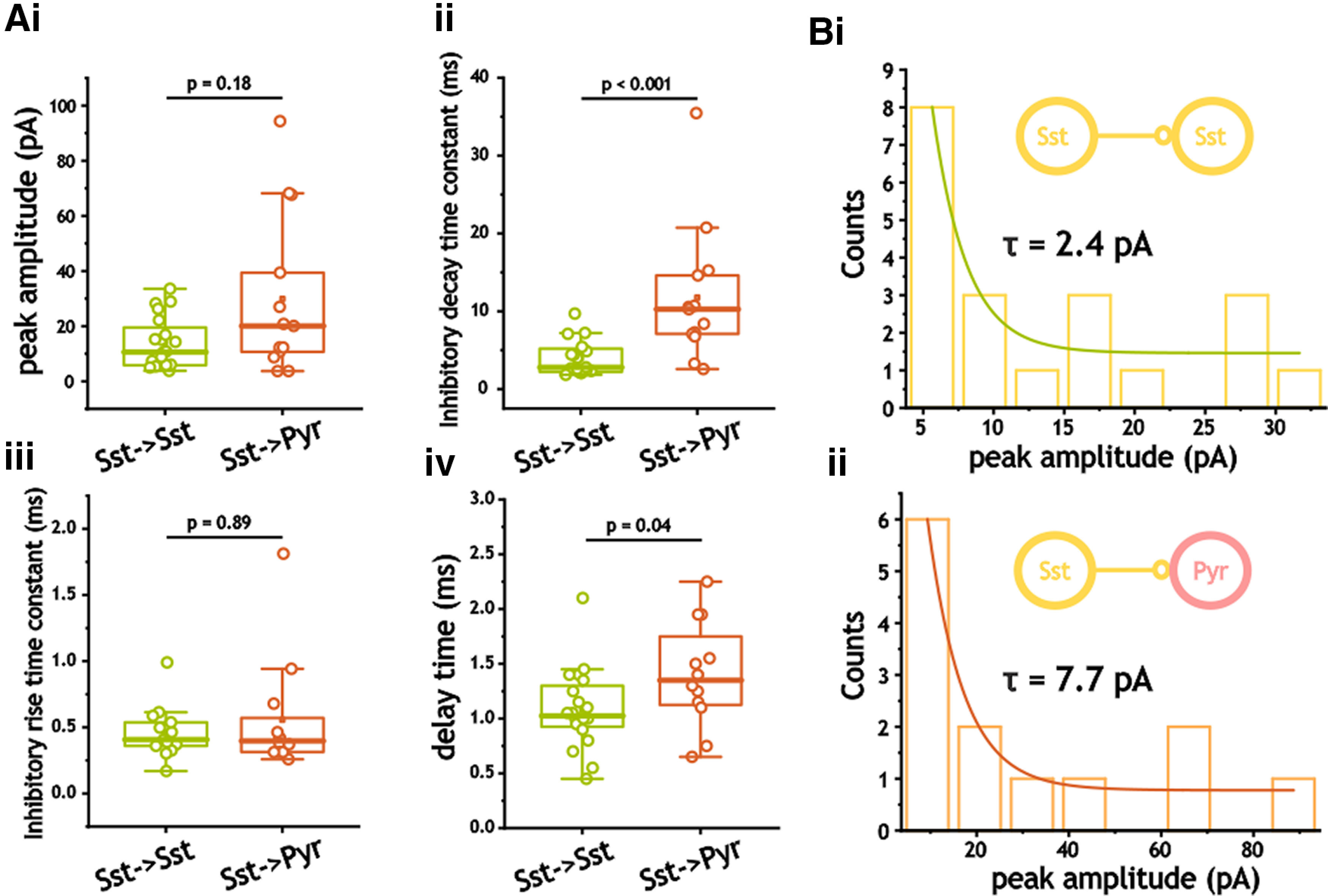
Synaptic inhibitory properties from Sst^+^ interneurons. ***Ai–iv***, Comparison of peak amplitude (***i***), inhibitory decay time constant (***ii***), inhibitory rise time constant (***iii***), and delay time (***iv***). ***Bi***, ***ii***, Distributions of inhibitory synaptic amplitude in Sst^+^ interneurons and pyramidal cells. Histograms were fit with a single exponential function.

As with Pvalb^+^ cells, inhibition from Sst^+^ cells onto pyramidal cells was significantly slower to decay than that between Sst^+^ cells [2.8 ms (Q1, 2.2; Q3, 5.2) vs 10.3 ms (Q1, 6.9; Q3, 14.9); *p* < 0.001, Mann–Whitney test; Fig. 5*Aii*). We also noted significantly longer delays in pyramidal cells (1.1 ± 0.14  vs 1.4 ± 0.08 ms; *p* = 0.04, Student's *t* test). Both peak amplitude and rise time constants, however, were not significantly different (*p* = 0.18 and *p* = 0.89; Mann–Whitney test; Fig. 5*Ai*,*iii*). Again, distributions of peak inhibitory amplitude in both SSt^+^ and pyramidal cells expressed non-Gaussian distributions (*p* < 0.001, Shapiro–Wilk and Lilliefors test) and could be fit with exponential functions (Fig. 5*Bi*,*ii*).

### Pvalb^+^ and Sst^+^ interneuron-based inhibition expresses different decay and delay times

**Figure 6.**
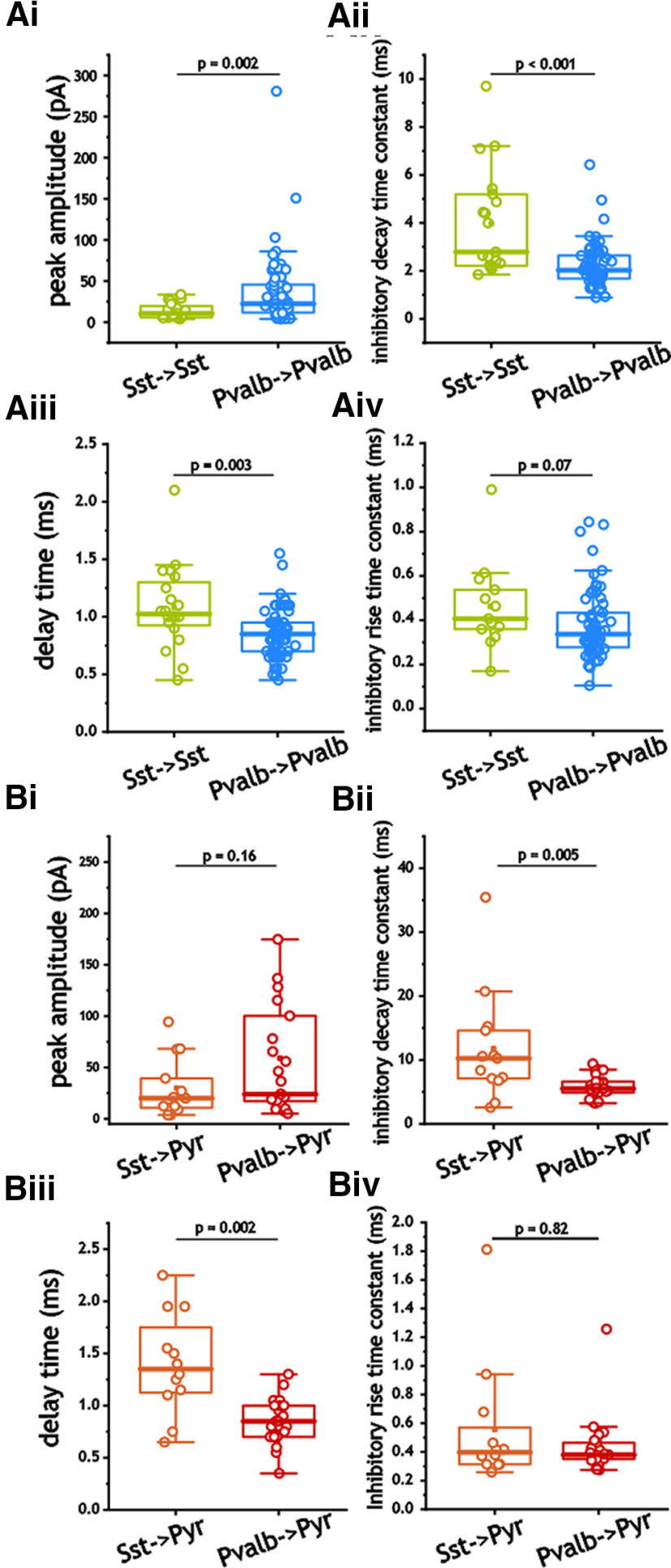
***Ai–Biv***, Synaptic inhibitory properties in interneurons (***Ai–iv***) and excitatory cells (***Bi–iv***). ***Ai–Biv***, Comparison of peak amplitude (***Ai***, ***Bi***), inhibitory decay time constant (***Aii***, ***Bii***), inhibitory rise time constant (***Aiii***, ***Biii***), and delay time (***Aiv***, ***Biv***) in synaptic connection in interneurons (***Ai–iv***) and excitatory cells (***Bi–iv***).

Comparison of inhibition in I_Sst_–I_Sst_ and I_Pvalb_–I_Pvalb_ pairs also indicated numerous differences. Inhibitory peak amplitude in I_Sst_–I_Sst_ pairs was significantly smaller (*p* = 0.002, Mann–Whitney test), with larger decay time constants (*p* < 0.001, Mann–Whitney test) and longer delays (*p* = 0.003, Mann–Whitney test) than those measured in I_Pvalb_–I_Pvalb_ pairs (Fig. 6*Ai–iv*). Similarly, we also noted differences between I_Sst_–E_Pyr_ and I_Pvalb_–E_Pyr_ pairs. These included larger decay time constants (*p* = 0.005, Mann–Whitney test; Fig. 6*Bii*) and delay times (*p* = 0.002, Mann–Whitney test; Fig. 6*Biii*) in I_Sst_–E_Pyr_ pairs.

### I–I Synapses express greater synaptic depression than I–E synapses

**Figure 7.**
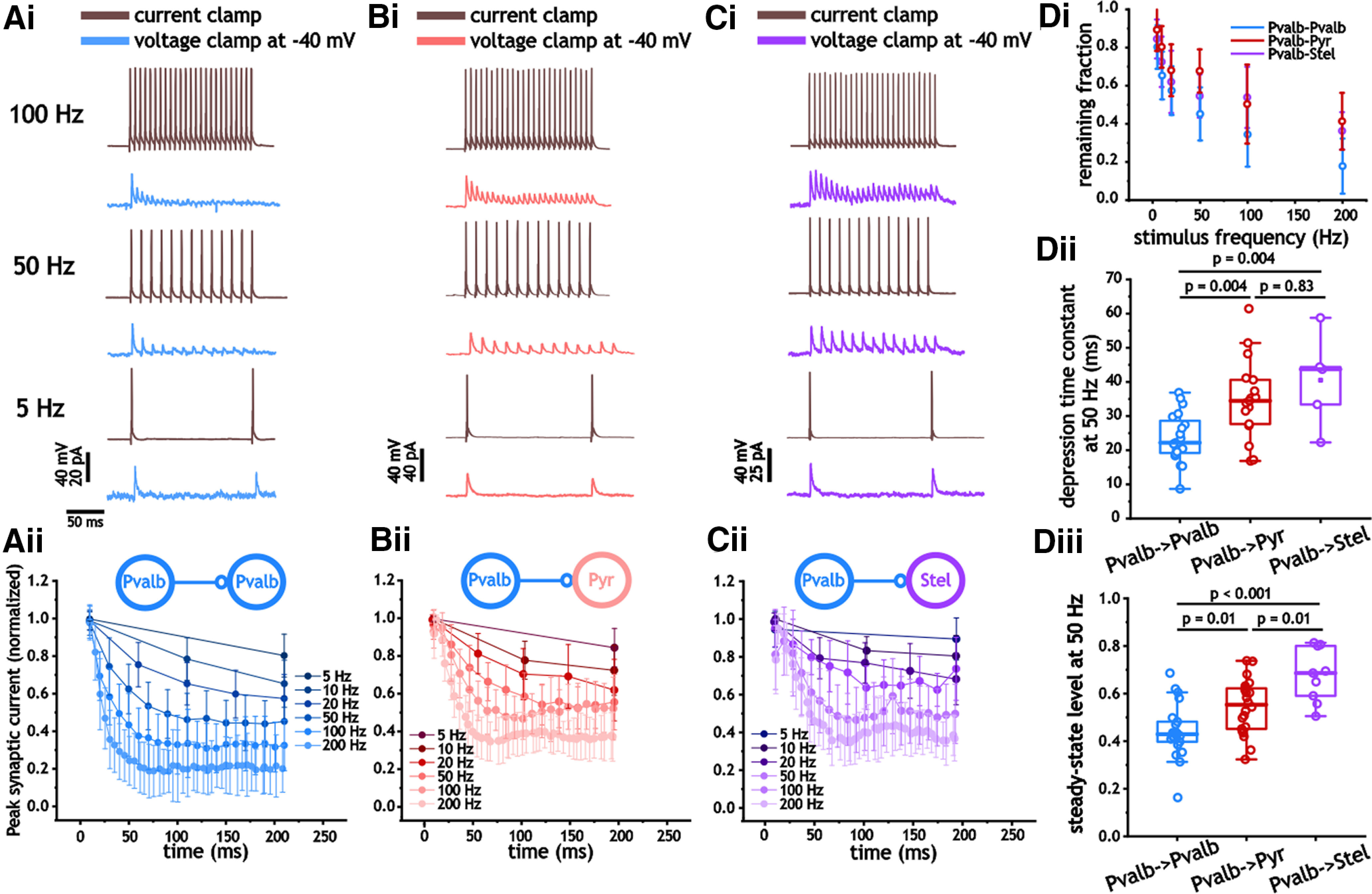
Synaptic inhibitory depression from Pvalb^+^ cells in Pvalb^+^, pyramidal, and stellate cells. ***Ai–Cii***, Measures of synaptic inhibitory depression in response to 200 ms presynaptic pulse trains between 5 and 200 Hz in Pvalb^+^ (***Ai***, ***ii***), pyramidal (***Bi***, ***ii***), and stellate (***Ci***, ***ii***) cells. ***Aii***, ***Bii***, ***Cii***, For each cell, synaptic inhibitory amplitude was normalized by the peak amplitude of the first response during the train. ***Di***, Plot of average normalized synaptic inhibitory amplitude at the end of different pulse trains. ***Di–iii***, Decay time constant associated with synaptic depression in response to 50 Hz pulse trains in Pvalb^+^, pyramidal, and stellate cells. Relative steady-state value of peak inhibitory synaptic amplitude in response to 50 Hz pulse trains in Pvalb^+^, pyramidal, and stellate cells.

Using 200-ms-long pulse trains between 5 and 200 Hz, we proceeded to measure the degree of synaptic depression at different stimulus frequencies. In both I_Pvalb_–I_Pvalb_ and I_Pvalb_–E synapses, depression was often observed at frequencies as low as 5–10 Hz (Fig. 7*Ai–Cii*). In all three cell types, synaptic depression increased as a function of stimulus frequency (Fig. 7*Di*). To compare differences in the depression, we measured the depression decay time constant and steady-state value at 50 Hz, a frequency in which depression was observed reliably in all three cells and reached a steady-state value within our stimulus time frame. As shown, the depression decay time constant and the steady-state value indicated faster and greater depression in I_Pvalb_–I_Pvalb_ synapses than in either type of I_Pvalb_–E connection (*n* = 61, *n* = 20, *n* = 12; *p* < 0.001, one-way ANOVA; Fig. 7*Dii*,*iii*), with I_Pvalb_–I_Pvalb_ pairs expressing a time constant of 20.6 ± 7.2 ms (vs 34.9 ± 11.7 and 40.5 ± 13.6 ms; *p* = 0.004, Bonferroni’s test; Fig. 7*Dii*) and a steady-state value at 50 Hz of 0.44 ± 0.11 (vs 0.55 ± 0.11 and 0.68 ± 0.11; *p* = 0.01 and *p* < 0.001, Bonferroni’s test; Fig. 7*Diii*).

**Figure 8.**
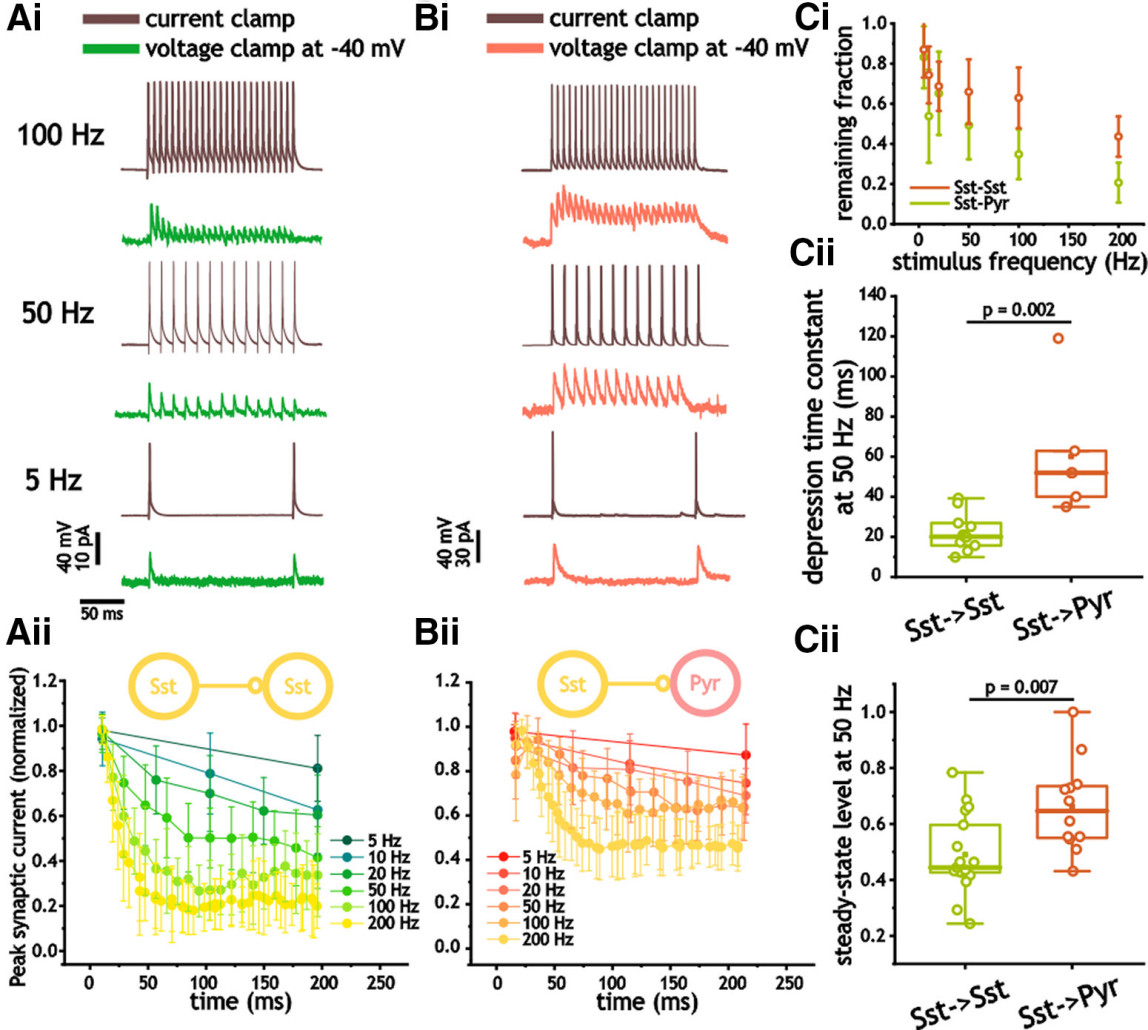
Synaptic inhibitory depression from Sst^+^ cells in Sst^+^ and pyramidal cells. ***Ai–Bii***, Measures of synaptic inhibitory depression in response to 200 ms presynaptic pulse trains between 5 and 200 Hz in Sst^+^ (***Ai***, ***ii***) and pyramidal (***Bi, ii***) cells. ***Aii***, ***Bii***, For each cell, synaptic inhibitory amplitude was normalized by the peak amplitude of the first response during the train. ***Ci***, Plot of average normalized synaptic inhibitory amplitude at the end of different pulse trains. ***Cii***, Decay time constant associated with synaptic depression in response to 50 Hz pulse trains in Pvalb^+^, pyramidal, and stellate cells. ***Ciii***, Relative steady-state value of peak inhibitory synaptic amplitude in response to 50 Hz pulse trains in Sst^+^ and pyramidal cells.

Using an identical stimulus range, we also measured the depression at I_Sst_–I_Sst_ and I_Sst_–E_Pyr_ synapses. Again, synaptic depression was observed at frequencies as low as 5–10 Hz. Like Pvalb^+^ cells, I_Sst_–I_Sst_ synapses depressed more quickly and to a greater extent than I_Sst_–E_Pyr_ synapses (Fig. 8*Ai–Cii*). The depression decay time constant in response to 50 Hz stimulus trains for I_Sst_–I_Sst_ pairs was 20.1 ms (Q1, 15.7; Q3, 26.9), which was significantly smaller than the 51.9 ms (Q1, 38.8; Q3, 76.9) measured in I_Sst_–E_Pyr_ synapses (*n* = 20, 13; *p* = 0.002, Mann–Whitney test; Fig. 8*Ci*). Steady-state levels at 50 Hz also differed, with I_Sst_–I_Sst_ pairs depressing to 0.44 (Q1, 0.42; Q3, 0.62) compared with 0.65 (Q1, 0.55; Q3, 0.74) in I_Sst_–E_Pyr_ pairs (*p* = 0.007, Mann–Whitney test; Fig. 8*Ciii*). Finally, there was no difference in the synaptic depression time constants (*p* = 0.55, Mann–Whitney test) and steady-state levels (*p* = 0.55, Mann–Whitney test) of synaptic depression in response to 50 Hz between I_Pvalb_–I_Pvalb_ and I_Sst_–I_Sst_ pairs.

### Modeling synaptic kinetics and short depression

**Figure 9.**
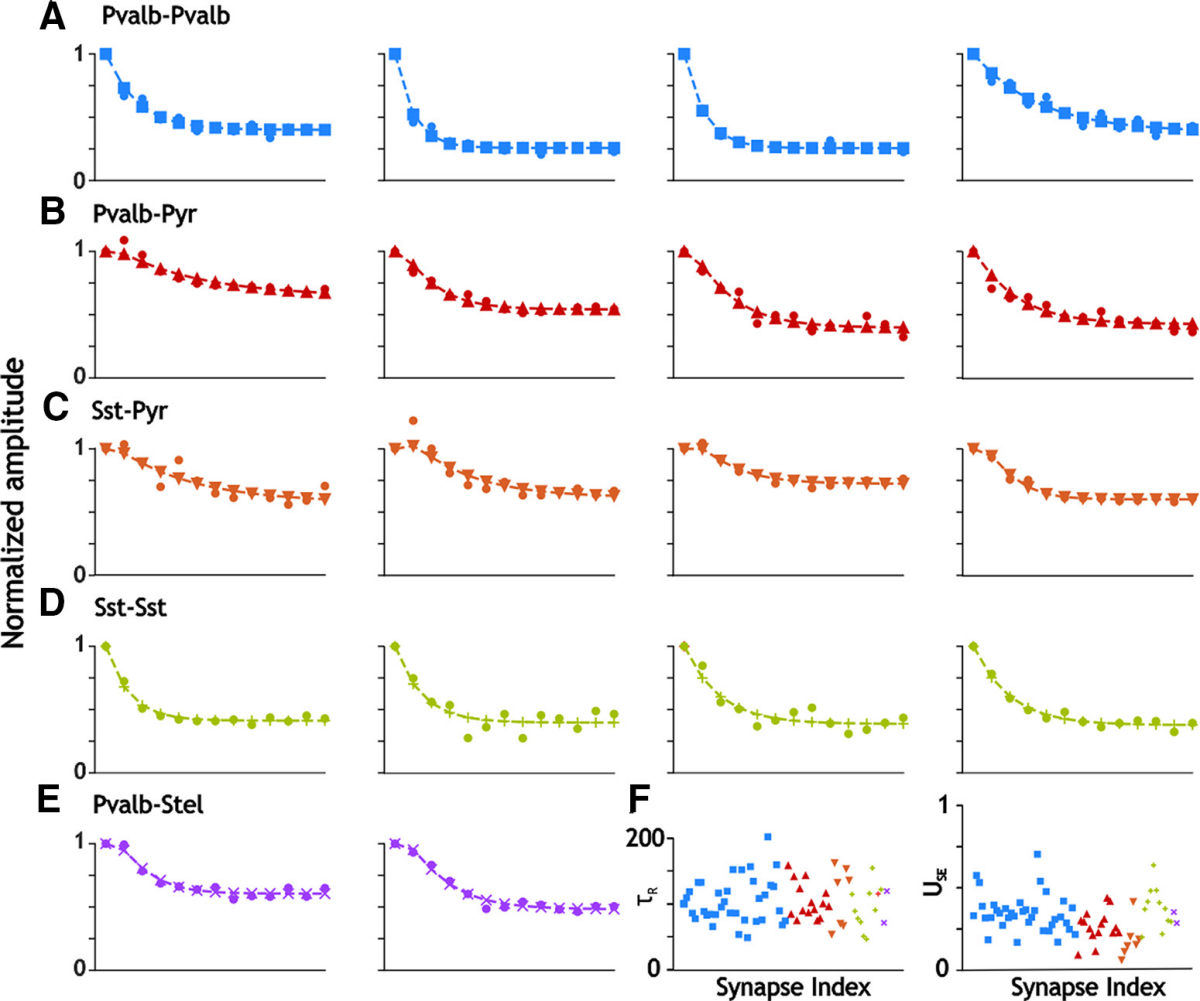
Modeling synaptic depression. The fits of inhibitory amplitude depression in response to 50 Hz trains of presynaptic stimulation are shown for selected examples for each synapse type. Data points are shown as filled circles. The values predicted by the best fit models are given by other shapes and colors. ***A***, Pvalb-Pvalb only includes depression. ***B***, Pvalb-Pyr includes both temporal summation and depression. ***C***, Sst-Pyr included both temporal summation and depression. ***D***, Sst-Sst includes only depression. ***E***, Pvalb-Stel included both temporal summation and depression. This was the smallest dataset, and only the two traces shown had a good enough fit according to the criterion given in Materials and Methods. ***F***, Parameter summary. Parameter values are coded by synapse type: Pvalb–Pvalb (blue squares), Pvalb–Pyr (red triangles), Sst–Pyr (upside down orange triangles), Sst–Sst (green plus signs), and Pvalb^+^–Stel (purple Xs). Left, Time constant for recovery from synaptic depression. Right, Fraction of available transmitter released by each presynaptic spike.

To examine whether our results could be framed using previous mechanism of synaptic depression, we used a model of short-term depression developed by Markram and Tsodyks (1996) to fit our measures of postsynaptic current decay and depression (Markram and Tsodyks, 1996; Stimberg et al., 2019). In Figure 9, we show the best fitting models of synaptic depression, as described in the Materials and Methods section. Because the synaptic decay time constants were much smaller for synapses between interneurons (Fig. 9*A*,*D*; I_Pvalb_–I_Pvalb_ and I_Sst_–I_Sst_, respectively), we were able to ignore temporal summation between successive postsynaptic currents and still obtain a good fit. In contrast, synapses onto excitatory cells were generally slow enough that including their previously measured synaptic decay constants in the model, as described in Materials and Methods, greatly improved the fit of the model (for I_Pvalb_–E_Pyr_, I_Sst_–E_Pyr_, and I_Pvalb_–E_Stel_, respectively; Fig. 9*B*,*C*,*E*). This is consistent with the observation that inhibitory currents decay more slowly on excitatory compared with inhibitory neurons (Ma et al., 2012). The summary panel in Figure 9*F* shows that the time constants for recovery from depression ranges from ∼50 to 150 ms, with no systematic differences in their values, as confirmed by the statistics in Table 1 (*p* = 0.89, one-way ANOVA). Most values of *U*_SE_ fall in the range 0.2–0.5, with some lower values (corresponding to less depression) associated with synapses onto pyramidal neurons (green and yellow symbols). Values, however, were not significantly different (*p* >0.05, Dunn’s test).

**Table 1:** Synaptic depression model parameter statistics

	Pvalb^+^–Pvalb^+^ (*n* = 36)	Pvalb^+^–Pyr (*n* = 15)	Pvalb^+^-Stel (*n* = 2)	Sst–Pyr (*n* = 7)	Sst–Sst (*n* = 11)
*U*_SE_	0.345 ± 0.019 (0.166, 0.702)	0.261 ± 0.023 (0.087, 0.433)	0.317 ± 0.024 (0.284, 0.350)	0.183 ± 0.040 (0.061, 0.415)	0.378 ± 0.37 (0.197, 0.636)
τ*_r_*	105.4 ± 5.5 (49.7, 202.9)	104.4 ± 6.6 (74.9, 158.8)	96.3 ± 17.0 (72.2, 120.4)	112.2 ± 15.9 (55.2, 163.8)	96.4 ± 9.4 (47.3, 155.5)

The mean value and the SEM are given for each parameter value, with *n* indicating the number of datasets that fit the *R*^2^ criterion given in the Materials and Methods and were included in Figure 9*F* and this table. Values in parentheses indicate the range of values.

## Discussion

In summary, we find that inhibition in I_Pvalb_–I_Pvalb_ and I_Pvalb_–E pairs in layer 2/3 mEC are stronger, faster, and more interconnected, both in term of synapses and gap junctions, than those measured in I_Sst_–I_Sst_ and I_Sst_–E_Pyr_ pairs. Further, for both Pvalb^+^ and Sst^+^ cells, inhibitory synaptic currents between interneuron pairs were faster to decay than in the excitatory cells; the inhibitory current decay time constant and delay time, however, were smaller and shorter, respectively, in Pvalb^+^ cells. Although we noted some minor differences, Pvalb^+^ inhibition onto pyramidal and stellate cells was generally similar. In contrast to previous measures in visual cortex (Pfeffer et al., 2013), we found significant evidence for inhibitory connections between Sst^+^ interneurons. Finally, inhibition between interneurons expressed faster and greater synaptic depression than those onto excitatory cells.

### Comparison with past measures of inhibitory synaptic currents

Like measures in neocortex (Beierlein et al., 2003) and hippocampus (Bartos et al., 2002), I_Pvalb_–I_Pvalb_ pairs expressed fast-decaying synapses that were strongly depressing. These cell pairs also contained a high prevalence of gap junction connections, albeit with a lower likelihood than those measured in other neocortical regions (Gibson et al., 1999; Hjorth et al., 2009). Similarly, we also noted gap junction connections in I_Sst_–I_Sst_ pairs, but these were far less frequent than in I_Pvalb_–I_Pvalb_ pairs. This contrasts with measures in neocortex in which I_Sst_–I_Sst_ pairs are highly interconnected through gap junctions (>65% of pairs; Amitai et al., 2002; Fanselow et al., 2008). Also, in contrast to neocortex (Pfeffer et al., 2013), we measured substantial synaptic connections between Sst^+^ neurons. These stark difference might arise, in part, from the large diversity within the Sst^+^ population, which likely includes at least four different subtypes of interneurons across different cortical regions and layers (Urban-Ciecko and Barth, 2016; Yavorska and Wehr, 2016). Our analysis of spike half-width shows that Sst^+^ cells share a similar range of variance as those in measured Pvalb^+^ cells. This suggests a narrowly defined population in layer 2/3 of the mEC.

Similar to CA1 (Bartos et al., 2002), we found that the inhibition decay time constants from Pvalb^+^ is target cell specific, with smaller decay time constants in Pvalb^+^ cells; this was also true for Sst^+^ interneurons. A key factor in determining the kinetics of GABA_A_ receptor-mediated inhibition is the subunit composition of the receptor (Fritschy and Brünig, 2003; Barberis et al., 2007). Faster decay times are associated with the presence of the α−1 and α−2 subunits (Barberis et al., 2007). Immunohistochemical work in layer 2 mEC indicates a greater prevalence of the α−1 subunit in Pvalb^+^ cells than in either reelin-positive (stellate) or calbindin-positive (pyramidal) excitatory cells (Berggaard et al., 2018). As a result, the faster decay time of inhibition can be explained by different subunit compositions that support a faster inactivation of the receptor in Pvalb^+^ cells.

### Interneuron role in synchrony and gamma oscillations

Fast-firing interneurons are generally assumed to underlie fast negative feedback that is critical to balancing network excitation and inhibition (Wehr and Zador, 2003; Okun and Lampl, 2008; Atallah and Scanziani, 2009; Isaacson and Scanziani, 2011). In addition, for cells in close proximity (<100 μm), nearly half of fast-firing interneurons are connected through gap junctions (Deans et al., 2001). These features support network synchrony, as well as the generation of fast gamma-frequency oscillations (Beierlein et al., 2000; Deans et al., 2001; Bartos et al., 2002; Mann and Paulsen, 2007; Atallah and Scanziani, 2009; Pastoll et al., 2013). Consistent with this function, we observed that Pvalb^+^ cells inhibition onto pyramidal cells was faster to decay and arrived with a smaller delay time than Sst^+^ cells. Pvalb^+^ cells also expressed a higher prevalence of gap junction connections, and a much higher degree of interconnectivity between Pvalb^+^ neurons.

In models, the unique kinetics and connectivity properties of fast-firing interneurons can also generate gamma oscillations using solely an inhibitory network (Bartos et al., 2002, 2007; Tiesinga and Sejnowski, 2009; Tikidji-Hamburyan et al., 2015; Keeley et al., 2017; Tikidji-Hamburyan and Canavier, 2020), with experimental support for this form oscillatory activity noted in the hippocampus and entorhinal cortex (Butler et al., 2016, 2018). In contrast, neocortical Sst^+^ interneurons *in vivo* do not correlate with oscillatory network activity (Kwan and Dan, 2012). Although differences in inputs are likely involved (Pfeffer et al., 2013; Yavorska and Wehr, 2016), the lower synaptic and gap junction connectivity that we observed may also contribute to lower synchrony levels.

Like Pvalb^+^ cells, the inhibition in Sst^+^ cells was faster to decay than that in excitatory neurons. The faster decay times of inhibition between interneurons, therefore, may serve roles independent of network synchrony and oscillations. For example, a longer inhibitory decay time onto excitatory targets may arise as a compensation mechanism for the larger membrane time constants of these cells relative to both Pvalb^+^ and Sst^+^ interneurons. The average firing rates of excitatory cells in layer 2 mEC are also typically lower than those in inhibitory cells (Frank et al., 2001), such that a proportional impact on spike rate requires longer-lasting inhibition. As a result, differences in the inhibition kinetics may serve to compensate for differences in the neurophysiology of the postsynaptic target.

### Kinetics of inhibition in pyramidal and stellate cells

Inhibition originating from Pvalb^+^ cell was generally very similar in pyramidal and stellate cells. The only small and significant difference was the greater degree of steady-state depression in pyramidal cells when synapses were driven at 50 Hz. This would appear to be consistent with the central role of Pvalb^+^ interneurons in shaping spatial selectivity of grid cells (Miao et al., 2017), which include both pyramidal and stellate cells (Domnisoru et al., 2013; Tang et al., 2014). Our results are also consistent with a recent comparison of Pvalb^+^ inhibition onto stellate and pyramidal cells along the dorsal–ventral axis (Grosser et al., 2021). The authors reported no difference in the amplitude, paired-pulse ratio, and connectivity likelihood between inhibition in stellate and pyramidal cells (Grosser et al., 2021). Nevertheless, past work has indicated stronger labeling for the slower α−3 GABA_A_ receptor subunit in stellate cells (Berggaard et al., 2018). Although we noted a tendency for inhibition to be slower in stellate cells, this difference did not reach significance.

### Synaptic depression in fast-firing interneurons

Parvalbumin is a Ca^2+^ buffer with slow kinetics that has been shown to mediate paired-pulse depression (PPD; Caillard et al., 2000). Although synapses between fast-firing neurons have not been as well studied as inhibition in excitatory cells, synapses between fast-firing neurons in hippocampal area CA3 (Kohus et al., 2016), and the dentate gyrus both exhibit short-term depression (Bartos et al., 2001). Similarly, synapses from Pvalb^+^ neurons in the medial septum diagonal band of Broca onto CA1 stratum oriens interneurons and from local hippocampal Pvalb^+^ interneurons onto CA1 pyramidal neurons both exhibit short-term depression (Yi et al., 2021). Previously, different subtypes of Pvalb^+^ CA1 pyramidal neurons were found to have different paired-pulse ratios (Maccaferri et al., 2000) in synapses onto principal cells, with the axoaxonic cells exhibiting more PPD than in basket cells and bistratified cells. In area CA3, the synapses of axoaxonic cells and Pvalb^+^ basket cells onto the principal cells both exhibit short-term depression; carbachol decreases the inhibitory synaptic current magnitude but eliminates or greatly reduces short-term depression (Szabó et al., 2010). Pvalb^+^ basket cells synapses onto their principal cell targets in the dentate gyrus also exhibit PPD (Kraushaar and Jonas, 2000). In the striatum, the synapses of fast-spiking interneurons onto medium spiny striatal neurons also exhibit short-term depression (Gittis et al., 2010). Synapses from fast-firing interneurons onto pyramidal cells in neocortex also exhibit short-term depression, albeit not as much as synapses between pyramidal cells (Galarreta and Hestrin, 1998). This study extends the evidence for short-term depression as a characteristic feature of Pvalb^+^ interneurons. However, the short-term depression observed here for Sst^+^ interneurons may not be as general; for example, in mouse layer four somatosensory cortex, synapses made by Sst^+^ cells depressed much less than those made by Pvalb^+^ cells, and have a late component of facilitation (Ma et al., 2012). Short-term synaptic plasticity may be an important mechanism that allows neurons to detect complex temporal structures by functioning as a memory of events in the past few hundred milliseconds (Motanis et al., 2018).

## Synthesis

Reviewing Editor: Mikko Airavaara, Helsingin yliopisto Decisions are customarily a result of the Reviewing Editor and the peer reviewers coming together and discussing their recommendations until a consensus is reached. When revisions are invited, a fact-based synthesis statement explaining their decision and outlining what is needed to prepare a revision will be listed below. The following reviewer(s) agreed to reveal their identity: Carl Lupica, Matthew Nolan. Note: If this manuscript was transferred from JNeurosci and a decision was made to accept the manuscript without peer review, a brief statement to this effect will instead be what is listed below.

Parvalbumin (PV) and somatostatin (SST) inhibitory interneurons represent the largest groups of this class of cells in the brain, and their activity both inhibits and synchronizes the activity of many neurons within the medial entorhinal cortex (mEC). This is important for the synchronization of mEC cells and their connections to downstream areas. Thus, they play an important role in cognition and other essential cortical processes. The investigators used whole cell recording of neurons in brain slices from mice bred to express tdTomato fluorescence in either SST or PV neurons to permit identification of these respective cell types using microscopy in brain slices. These in vitro electrophysiological recordings included measurement of membrane physical properties, gap junction connectivity, and synaptic current properties from a large number of pairs of synaptically coupled SST-SST, PV-PV neurons. Additionally, connections between these cells and local excitatory neurons were studied. The main findings are that inhibition between pairs of PV-PV interneurons and PV-excitatory cells (EC) in layer 2/3 of the medial entorhinal cortex (mEC) is stronger, faster, and PV-PV neurons more interconnected by synapses and gap junctions, than SST-SST pairs or SST-EC pairs. They also report that IPSCs among parvalbumin and somatostatin cells decay more quickly than IPSCs observed in excitatory cells. Moreover, inhibition among interneurons was kinetically faster and stronger than inhibition of ECs in mEC. The detailed analysis of these measures included biophysical and connection properties among these cells.

The study is highly rigorous, includes many cells and many paired recordings. It is possible that this work could be used as a reference for connectivity parameters to be fed into mEC circuit modeling studies. In general, the studies appear to be expertly conducted and the data properly interpreted.

1. There is a concern that that the work may be useful to a smaller more specialized neuroscience audience. Thus, it would be suggested that the authors revise the manuscript in terms of significance of the study.

2. The authors indicate that there is a large diversity within the SST neuron population that includes at least 4 different subtypes of interneurons. However, there is not much discussion as to how these subgroups differ. Is it possible that these subgroups might be identified biophysically or through anatomical means and this information could be used in the present analysis?

3. Line 188-191. I understand why the authors have estimated synaptic response kinetics and latencies from averaged responses, but this may introduce systematic errors as its difficult to perfectly align the synaptic responses when calculating an average. This could be a particular issue for comparisons of rise-time constants. It would be good if this approach can be validated for a subset of connections by comparison to kinetic and latency values estimated from individual responses and then averaged.

4. Line 262, “To detect synapses...”. Because spikes propagated via gap junctions can look like a chemical PSP, it would be helpful here to explain how a chemical synaptic response is distinguished from a gap junction mediated synaptic response. As presented it seems feasible that at least some of the bidirectional responses could by electrical rather than chemical synaptic responses.

5. In the Results section its unclear whether the recorded pyramidal neurons are located in layer 2 or layer 3. If both then the analyses should distinguish between pyramidal neurons in each layer.

6. Lines 62-62, “Fast-firing interneurons also provide the sole synaptic communication path between stellate cells, which lack recurrent excitatory connection", should cite Couey et al., 2013 as well as the Pastoll et al. study.

7. Methods should provide an experimentally determined estimate of the liquid junction potential.

8. As well as reporting the probability of gap junction connections in figure 1, it would be great to also show the steady-state coupling coefficients.

9. Line 333, “16/59 pairs", are these Sst-Sst pairs?

10. The methods should indicate the dorsoventral location of the recorded cells.

## Author Response

Response:

We thank the reviewer for the comments. Below we have addressed the reviewer's concerns. All changes to the text are indicated in red. We have included a figure in this response to address concern # 3.

Synthesis Statement for Author (Required):

Parvalbumin (PV) and somatostatin (SST) inhibitory interneurons represent the largest groups of this class of cells in the brain, and their activity both inhibits and synchronizes the activity of many neurons within the medial entorhinal cortex (mEC). This is important for the synchronization of mEC cells and their connections to downstream areas. Thus, they play an important role in cognition and other essential cortical processes. The investigators used whole cell recording of neurons in brain slices from mice bred to express tdTomato fluorescence in either SST or PV neurons to permit identification of these respective cell types using microscopy in brain slices. These in vitro electrophysiological recordings included measurement of membrane physical properties, gap junction connectivity, and synaptic current properties from a large number of pairs of synaptically coupled SST-SST, PV-PV neurons. Additionally, connections between these cells and local excitatory neurons were studied. The main findings are that inhibition between pairs of PV-PV interneurons and PV-excitatory cells (EC) in layer 2/3 of the medial entorhinal cortex (mEC) is stronger, faster, and PV-PV neurons more interconnected by synapses and gap junctions, than SST-SST pairs or SST-EC pairs. They also report that IPSCs among parvalbumin and somatostatin cells decay more quickly than IPSCs observed in excitatory cells. Moreover, inhibition among interneurons was kinetically faster and stronger than inhibition of ECs in mEC. The detailed analysis of these measures included biophysical and connection properties among these cells.

The study is highly rigorous, includes many cells and many paired recordings. It is possible that this work could be used as a reference for connectivity parameters to be fed into mEC circuit modeling studies. In general, the studies appear to be expertly conducted and the data properly interpreted.

1. There is a concern that that the work may be useful to a smaller more specialized neuroscience audience. Thus, it would be suggested that the authors revise the manuscript in terms of significance of the study.

Response:

We have now emphasized that we found connections between Sst+ cells. Previous work in visual cortex indicated that connections between Sst+ cells did not exist. We now place greater focus on this disparity as it indicates that a canonical model based on visual cortex does not apply across different cortical regions.

We revised lines 18-20 in the abstract to state that “we found that, unlike neocortical measures, Sst+ cells inhibit each other, albeit with a lower probability than Pvalb+ cells (18% versus 36% for unidirectional connections).”

We revised lines 85-85 in the introduction to read “Specifically, measures in visual cortex indicate a lack of synaptic connections between Sst+ interneurons (Pfeffer et al., 2013). “

We added this sentence to lines 111-112 of the significance statement “In contrast to neocortical measures, we find substantial connections between Sst+ interneurons.”

The results echo this point in lines 441-442: “In contrast to previous measures in visual cortex (Pfeffer et al., 2013), we found significant evidence for inhibitory connections between Sst+ interneurons.”

2. The authors indicate that there is a large diversity within the SST neuron population that includes at least 4 different subtypes of interneurons. However, there is not much discussion as to how these subgroups differ. Is it possible that these subgroups might be identified biophysically or through anatomical means and this information could be used in the present analysis?

Response:

Our comment is based on reviews of Sst+ neurons across different cortical regions. The reviews cover different cortical regions and layers. In our data set, most Sst+ neurons had features similar to Pvalb+ neurons in that they were relatively fast firing, albeit with slightly wider spikes and larger membrane time constants. In some cases, they also expressed more spike frequency adaptation than Pvalb+ cells. As shown in figure 4Aii, the distribution of spike half-widths for Sst+ neurons was normal (as determined using a Kolmogorov Smirnov test for large n values) and had a relatively tight distribution and small coefficient of variation (0.28), which was similar to that of Pvalb+ cell spike half-widths (0.21). Together this suggests a relatively homogeneous population in terms of spike width. The discrepancy between our results and those summarized in the reviews we cite stem from us only measuring Sst+ neurons in layer 2/3 mEC, as opposed to the reviews that focused on Sst+ across cortical layers and regions. We now emphasize that previous reviews cite diversity within Sst+ neurons across the brain, but not necessarily within a layer of a specific region. Finally, due to the lower number of connections, breaking down the Sst+ population into distinct subtypes leads to a loss of statistical power. For these reason, we did not breakdown Sst+ or Pvalb+ cells into sub categories.

We clarified this issue in lines 455-460 of the results: “Also, in contrast to neocortex (Pfeffer et al., 2013), we measured substantial synaptic connections between Sst+ neurons. These stark difference might arise, in part, from the large diversity within the Sst+ population, which likely includes at least 4 different subtypes of interneurons across different cortical regions and layers (Urban-Ciecko and Barth, 2016; Yavorska and Wehr, 2016). Our analysis of spike half-width shows that SST+ cells share a similar range of variance as those in measured Pvalb+ cells. This suggests a more narrowly defined population in layer 2/3 of the mEC that compared to other regions. “

3. Line 188-191. I understand why the authors have estimated synaptic response kinetics and latencies from averaged responses, but this may introduce systematic errors as its difficult to perfectly align the synaptic responses when calculating an average. This could be a particular issue for comparisons of rise-time constants. It would be good if this approach can be validated for a subset of connections by comparison to kinetic and latency values estimated from individual responses and then averaged.

Response:

Our motivation for averaging was that for a majority of the synaptic connections had individual responses that were small and near the detection threshold; without averaging responses, the synaptic response was often not detected using a single trial. To address the concern, however, we have added the distribution of individual fits and delays to that of the mean response for 3 examples were the IPSC amplitude was large (>50 pA) in PV-PV connections; in these examples, trace fits and measures of delay were possible on most trials. As indicated in the figure below, the time constant and delay of the mean response was close to the middle of the trial-to-trial distribution for both measures (always within the interquartile range).

Based on the data shown in Response Figure 1, we added these sentences to lines 196-199 of the methods: “In a subset of recordings, where individual responses were large, we compared the averaged response to the distribution across trials. In this data set, mean responses sat near the center of the trial-to-trial distribution, suggesting that averaging did not impact our estimates of rise kinetics and delay.”

4. Line 262, “To detect synapses...”. Because spikes propagated via gap junctions can look like a chemical PSP, it would be helpful here to explain how a chemical synaptic response is distinguished from a gap junction mediated synaptic response. As presented it seems feasible that at least some of the bidirectional responses could by electrical rather than chemical synaptic responses.

Response:

In a subset of cells, we used a square current pulse that did not generate spikes to detect gap junctions. In some pairs, however, we used the action potential as well. Chemical synapses were distinguished due to the latency and polarity of the current. In terms of latency, gap junction-mediated current tracked the action potential with no discernable latency. In terms of polarity, at -40 mV, gap junction-mediated currents were inward (negative), while inhibitory synaptic connections were outward (positive).

For clarity, we added this to lines 281-283 of the results: “Using a step depolarization between 10 and 20 mV in one cell, we measured the strength of gap junction connections between Pvalb+ cells as the ratio of the two voltage responses. This value was 0.03 [Q1 0.02 Q3 0.06] across 17 measured pairs.”

5. In the Results section its unclear whether the recorded pyramidal neurons are located in layer 2 or layer 3. If both then the analyses should distinguish between pyramidal neurons in each layer.

Response:

Pyramidal cells were from layer 2. This is now indicated throughout the revised manuscript, including lines 26-27 of the abstract and lines 259-261 of the results.

6. Lines 62-62, “Fast-firing interneurons also provide the sole synaptic communication path between stellate cells, which lack recurrent excitatory connection", should cite Couey et al., 2013 as well as the Pastoll et al. study.

Response:

We now cite the Couey et al study in line 68 of the introduction, as well as the Pastoll et al study..

7. Methods should provide an experimentally determined estimate of the liquid junction potential.

Response:

We now indicate our liquid junction potential by adding this sentence to line 181 of the methods:

"A measured junction potential of ∼11 mV was not subtracted from recordings.”

8. As well as reporting the probability of gap junction connections in figure 1, it would be great to also show the steady-state coupling coefficients.

Response:

We have now provided this for 17 pairs for Pvalb+ cells. As stated in point 4 above, we added this to lines 286-289 of the results: “Using a step depolarization between 10 and 20 mV in one cell, we measured the strength of gap junction connections between Pvalb+ cells as the ratio of the two voltage responses. This value was 0.03 [Q1 0.02 Q3 0.06] across 17 measured pairs.”

9. Line 333, “16/59 pairs", are these Sst-Sst pairs?

Response:

Yes, this is now indicated in line 340: “we found that 16/59 Sst+ pairs expressed a connection “

10. The methods should indicate the dorsoventral location of the recorded cells.

Response:

This is now indicated in the methods section in lines 181-182 of the methods section

Recordings were taken from slices between 3.2 mm and 4.3 mm from the dorsal surface of the brain.
